# The impact of CYP2D6-predicted phenotype on tamoxifen treatment outcome in patients with metastatic breast cancer

**DOI:** 10.1038/sj.bjc.6605800

**Published:** 2010-08-10

**Authors:** L A Lammers, R H J Mathijssen, T van Gelder, M J Bijl, A-J M de Graan, C Seynaeve, M A van Fessem, E M Berns, A G Vulto, R H N van Schaik

**Affiliations:** 1 Department of Hospital Pharmacy, Erasmus MC, University Hospital, P.O. Box 2040, 3000 CA, Rotterdam, The Netherlands; 2 Department of Medical Oncology, Erasmus MC, University Hospital, P.O. Box 2040, 3000 CA, Rotterdam, The Netherlands; 3 Department of Internal Medicine, Erasmus MC, University Hospital, P.O. Box 2040, 3000 CA, Rotterdam, The Netherlands; 4 Department of Epidemiology, Erasmus MC, University Hospital, P.O. Box 2040, 3000 CA, Rotterdam, The Netherlands; 5 Department of Clinical Chemistry, Erasmus MC, University Hospital, P.O. Box 2040, 3000 CA, Rotterdam, The Netherlands

**Keywords:** tamoxifen, CYP2D6, pharmacogenetics, metastatic breast cancer, phenotype

## Abstract

**Background:**

Cytochrome P450 2D6 (CYP2D6) has a crucial role in the metabolic conversion of tamoxifen into the active metabolite endoxifen. In this cohort study, the effect of CYP2D6-predicted phenotype, defined as the combined effect of CYP2D6 genetic variation and concomitant use of CYP2D6-inhibiting medication, on time to breast cancer progression (TTP) and overall survival (OS) in women who use tamoxifen for metastatic breast cancer (MBC) was examined.

**Methods:**

We selected patients treated with tamoxifen (40 mg per day) for hormone receptor-positive MBC from whom a blood sample for pharmacogenetic analysis (CYP2D6*3, *4, *5, *6, *10 and *41) was available. Patient charts (*n*=102) were reviewed to assess TTP and OS, and to determine whether CYP2D6 inhibitors were prescribed during tamoxifen treatment.

**Results:**

OS was significantly shorter in patients with a poor CYP2D6 metaboliser phenotype, compared with extensive metabolisers (HR=2.09; *P*=0.034; 95% CI: 1.06–4.12). Co-administration of CYP2D6 inhibitors alone was also associated with a worse OS (HR=3.55; *P*=0.002; 95% CI: 1.59–7.96) and TTP (HR=2.97; *P*=0.008; 95% CI: 1.33–6.67) compared with patients without CYP2D6 inhibitors.

**Conclusion:**

CYP2D6 phenotype is an important predictor of treatment outcome in women who are receiving tamoxifen for MBC. Co-administration of CYP2D6 inhibitors worsens treatment outcome of tamoxifen and should therefore be handled with care.

## Introduction

Breast cancer is the most common type of cancer among women worldwide with nearly 1.2 million new diagnoses each year (Kamangar *et al*, 2006). Tumour expression of the oestrogen receptor (ER) and/or progesterone receptor (PR) has an important role in the choice for systemic treatment. In ER-positive and/or PR-positive breast cancer, anti-oestrogenic therapy has proven efficacy in both the adjuvant and metastatic setting (Riggs and Hartmann, 2003).

Tamoxifen is a so-called selective oestrogen-receptor modifier (Mortimer *et al*, 2007) with potent anti-oestrogenic action, which competes with oestrogen at the ER, resulting in the inhibition of growth of ER-positive breast cancer cells (Riggs and Hartmann, 2003). The hormonal receptor status therefore is an important predictive factor for the response of tamoxifen in breast cancer (Fisher *et al*, 2005). Tamoxifen is considered a pro-drug, metabolised by phase I enzymes (cytochrome P450 (CYP2D6) iso-enzymes 3A4, 2D6 and others) into metabolites including *N*-desmethyl-tamoxifen (NDM-TAM), 4-hydroxy-tamoxifen (4-OH-TAM) and finally the more potent metabolite 4-hydroxy-NDM-TAM(endoxifen). Both 4-OH-TAM and endoxifen are known to have a 30–100 times higher anti-oestrogen activity than tamoxifen. However, on average, plasma concentrations of endoxifen are approximately 10 times higher than those of 4-OH-TAM, making endoxifen the most potent metabolite of tamoxifen (Borges *et al*, 2006). In addition, endoxifen was recently proven to be the most potent metabolite in tamoxifen therapy with respect to the growth inhibition of breast cancer cells (Wu *et al*, 2009). Cytochrome P450 2D6 has a crucial role in the formation of endoxifen (Goetz *et al*, 2008). The activity of this enzyme is partly determined by the presence of polymorphisms in the *CYP2D6* gene, resulting in differences in the metabolising capacity. On the basis of CYP2D6-metabolising capacity, the population can be divided into four phenotypes. Ultrarapid metabolisers have duplicated *CYP2D6* genes, and thus express excessive amounts of functional enzyme. Carriers of two functional alleles exhibit normal enzyme activity and are classified as extensive metabolisers (EMs). Patients with one functional and one non-functional allele or two decreased activity alleles are referred to as intermediate metabolisers (IMs). Individuals with two non-functional alleles of the gene lack CYP2D6 enzymatic activity and, therefore, are classified as poor metabolisers (PMs). In Caucasians, CYP2D6*4 allele is the most common non-functional allele, having an allele frequency of about 20% (Bradford, 2002). Other dysfunctional alleles that are (less frequently) seen in the Caucasian population are CYP2D6*3, CYP2D6*5 and CYP2D6*6, with allele frequencies of 1–2%, 2–7% and 1%, respectively, (Bradford, 2002).

A few years ago, an association between CYP2D6 genotype and clinical outcome in tamoxifen-treated breast cancer patients was described by Goetz *et al* (2005). Postmenopausal women with the homozygous CYP2D6*4 genotype (PM) using tamoxifen tend to have a higher risk of breast cancer recurrence than EMs. This finding was confirmed by Schroth *et al* (2007) in a retrospective study among patients using tamoxifen as an adjuvant therapy and by Newman *et al* (2008). Women with the dysfunctional CYP2D6 alleles *4, *5, *10 and *41 had an increased risk of breast cancer recurrence and worse relapse-free survival rates. More recently, a multicenter study including retrospectively and prospectively collected patient data also confirmed an association between CYP2D6 variation and clinical outcome in women receiving adjuvant tamoxifen (Schroth *et al*, 2009). However, also negative results or even opposite findings have been published (Nowell *et al*, 2005; Wegman *et al*, 2005, 2007) and recently reviewed (Dezentje *et al*, 2009; Hoskins *et al*, 2009).

Besides the CYP2D6 genotype, concomitant use of CYP2D6-inhibiting medication may also be an important factor influencing the clinical outcome of tamoxifen treatment. Important CYP2D6-inhibiting drugs are, for instance, selective serotonin reuptake inhibitors (SSRIs), which may be used in breast cancer patients to treat depression or hot flashes; a common side effect of tamoxifen (Loprinzi *et al*, 2000). Goetz *et al* (2007)have shown that poor CYP2D6 metabolisers, due to genetic polymorphisms and/or CYP2D6-inhibiting medication (further defined as ‘predicted phenotype’), using tamoxifen as adjuvant therapy do have a significantly shorter time to breast cancer recurrence than EMs. Furthermore, a presented review of medical records involving breast cancer patients treated with tamoxifen showed that concomitant use of CYP2D6-inhibiting medication increased the risk of breast cancer recurrence (Aubert *et al*, 2009). In addition, in a recently published cohort study, paroxetine use during tamoxifen treatment was associated with an increased risk of death from breast cancer (Kelly *et al*, 2010). In contrast, Dezentje *et al* (2010)reviewed Dutch pharmacy data and reported opposite findings. They found no evidence that CYP2D6 inhibitors increase the risk of breast cancer recurrence.

These available results clearly warrant further research to test the hypothesis whether CYP2D6 phenotype is associated with the clinical outcome in tamoxifen-treated breast cancer patients. Moreover, studies in patients with metastatic disease are very scarce. To our knowledge, so far only one study reported on the effect of CYP2D6 genotype in a small number of Asian breast cancer patients using tamoxifen for metastatic disease evaluating the association of the dysfunctional allele CYP2D6*10/*10 (commonly occurring in the Eastern people in contrast to Caucasians) with clinical outcome (Lim *et al*, 2007). It was found that the PM phenotype based on genotype was associated with a significantly shorter time to progression (TTP). A correlation between CYP2D6 phenotype, and clinical outcome in metastatic breast cancer (MBC) patients of Caucasian origin has not been described yet. Therefore, the present study aimed to investigate the effect of CYP2D6-predicted phenotype, defined as the combined effect of the most common CYP2D6 genetic polymorphisms in Caucasians (*3, *4, *5, *6, *10 and *41) and concomitant use of CYP2D6-inhibiting medication, on TTP and overall survival (OS) in female MBC patients treated with tamoxifen.

## Materials and methods

### Patients and study design

A consecutive series of patients treated with tamoxifen for breast cancer at the Daniel den Hoed cancer centre of the Erasmus MC University hospital, were sampled for pharmacogenetic analysis between 2000 and 2008. From that dataset we selected all patients with MBC, having a positive hormone receptor status (ER and/or PR), and receiving a tamoxifen dose of 40 mg per day. In The Netherlands, 40 mg per day is the standard tamoxifen dose for MBC patients compared with 20 mg per day in the adjuvant setting. As the terminal elimination half-life of tamoxifen for a single dose is 5–7 days, and the time to reach steady state plasma concentrations is 3–4 weeks, an observational period of 1 month may be necessary before the effect of hormonal therapy can be seen (Morello *et al*, 2003). Therefore, participants using tamoxifen for <30 days were also excluded. Patients started with tamoxifen between 1986 and 2008. This study was approved by the local medical ethics board (study numbers AZR00/168A and MEC02/1002), and all patients gave written informed consent. Patient charts were reviewed to record the following data: age at start of tamoxifen therapy for metastatic disease, race, ER and PR status, treatments before tamoxifen therapy, number and location of metastatic sites, CYP2D6-inhibiting co-medication, TTP and OS.

Participants were monitored from the start of first tamoxifen prescription for MBC until death, or until the end of the study period (July 2009), whichever came first. Time to progression was defined as the time from first tamoxifen prescription for MBC to the documentation of progression, which was assessed by standard RECIST criteria (Hayward *et al*, 1977). Overall survival was defined as the time from first tamoxifen prescription for MBC to death due to any cause.

Patient charts were also reviewed to determine whether the following known CYP2D6 inhibitors were co-administered during the time that tamoxifen was used for metastatic disease: fluoxetine, paroxetine and bupropion (all strong inhibitors), duloxetine and terbinafine (moderate inhibitors), amiodarone, cimetidine, citalopram and sertraline (all weak inhibitors) (Flockhart, 2007).

### CYP2D6 genotyping

All patients were genotyped for the CYP2D6*3, *4, *5 and *6 polymorphisms, which will detect over 95% of CYP2D6 PMs and also for the CYP2D6 polymorphisms *10 and *41 that are associated with reduced enzyme activity. The source of genomic DNA was EDTA blood and the genotyping was done using Taqman allelic discrimination assays on the ABI Prism 7000 (Applied Biosystems, Nieuwerkerk aan den IJssel, the Netherlands) Sequence detection system. Primers and probes were designed by Applied Biosystems using their Assay-by-Design service. Polymerase chain reactions (PCR) were carried out in a reaction volume of 10.0 *μ*l, containing assay-specific primers, allele-specific Taqman MGB probes (Applied Biosystems), Abgene Absolute QPCR Rox Mix (Applied Biosystems) and genomic DNA (1 ng). The thermal profile consisted of an initial denaturation step at 95°C for 15 min, followed by 40 cycles of denaturation at 92°C for 15 s and annealing and extension at 60°C for 1 min. The *5 was determined using long range PCR on 20 ng genomic DNA using primers 5′-CACACCGGGCACCTGTACTCCTCA-3′, 5′-CAGGCATGAGCTAAGGCACCCAGAC-3′, 5′GTTATCCCAGAAGGCTTTGCAGGCTTCA-3′ and 5′-GCCGACTGAGCCCTGGGAGGTAGGTA-3′ with PCR profile 7 min 94°C, followed by 35 cycles of 1 min 94°C, 1 min 65°C annealing and 5 min 68°C elongation using rTth DNA polymerase (Applied Biosystems). PCR products were analysed on an EtBr gel. Presence of a *CYP2D6* gene deletion (*5) can be observed by the presence of a 3.5 kb fragment. A 5.1 kb fragment will always be amplified, and serves as an internal control. Genotypes were scored through measuring allele-specific fluorescence using the SDS 2.2.2 software for allelic discrimination (Applied Biosystems).

### CYP2D6 phenotyping

On the basis of CYP2D6 genotype in combination with concomitant use of CYP2D6-inhibiting medication, patients were classified into three phenotype groups (see Table 2). As an observational period of 3 or even 6 months may be necessary before the effect of hormonal therapy could be seen, concomitant CYP2D6 inhibitor use was defined as a minimum of 6 months overlap between tamoxifen and the CYP2D6 inhibitor (Muss *et al*, 1994). Women without a dysfunctional (CYP2D6*3, *4, *5 or *6) allele and who were not using a CYP2D6 inhibitor for at least 6 months (or until tamoxifen was stopped) were defined as EMs. Intermediate metabolisers (i) carry *CYP*2D6*10 or *41 alleles either homozygous or in combination with a dysfunctional allele or (ii) were heterozygous for the CYP2D6*3, *4, *5 or *6 allele (*3/wt, *4/wt, *5/wt or *6/wt) and did not use a CYP2D6 inhibitor or (iii) had no dysfunctional alleles but were using a weak or moderate CYP2D6 inhibitor. Women classified as PMs had (i) two dysfunctional alleles (for example, CYP2D6*3/*3, *3/*4 or *4/*4), or (ii) one dysfunctional allele (CYP2D6*3/wt, *4/wt, *5/wt or *6/wt) with concurrent use of a moderate CYP2D6 inhibitor or (iii) a functional genotype (wt/wt) with co-administration of a strong CYP2D6 inhibitor (Goetz *et al*, 2007).

### Statistical analysis

Deviations from Hardy–Weinberg equilibrium and differences in allele frequencies of the CYP2D6*3, *4, *5, *6, *10 and*41 alleles were analysed using *χ*^2^-tests. The effect of CYP2D6 genotype, CYP2D6-inhibiting co-medication and CYP2D6 phenotype (EM, IM, PM) on TTP and OS was assessed using Cox proportional hazards models. Analyses were adjusted for age. Confounders were adjusted for in the analysis if they caused a change in the point estimate of more than 10 percent. Kaplan–Meier estimates and log-rank test were used in univariate analysis of TTP and OS. The 5% cut-off level was chosen as significance level. All analyses were carried out using SPSS software (version 15.0, Chicago, IL, USA).

## Results

Of the 116 patients treated with tamoxifen for MBC enroled, 104 patients had a positive hormone receptor status. Two patients discontinued tamoxifen treatment within 30 days and were excluded from the analysis. The characteristics of the 102 patients treated with tamoxifen for MBC that met the inclusion criteria are described in Table 1. Patients started with tamoxifen for metastatic disease between 1986 and 2008. CYP2D6 genotype was determined in 99 of these patients. In our population, the allele frequencies of the CYP2D6*3, *4, *5, *6, *10 and*41 alleles were 3.5, 21.7, 1.5, 1.0, 1.5 and 6.6%, respectively, which is in line with earlier published data (Bradford, 2002). Genotype distributions were in Hardy–Weinberg Equilibrium (CYP2D6*3, *4, *5, *6, *10 and *41: *χ*^2^=0.46; *P*=0.25). Co-administration of CYP2D6 inhibitors (paroxetine, fluoxetine, sertraline and citalopram) occurred in 6.9% of patients (*N*=7, mean duration of co-administration: 11.5 months; range: 6 months to 1.6 years).

On the basis of CYP2D6-predicted phenotype definition, 48.5% of the patients were classified as EMs, 38.4% as IMs and 13.1% as PMs (Table 2). Patients used tamoxifen for a mean period of time of 2.8 years (range: 1.6 months to 17 years); being the longest (3.0 years) in the EMs and the shortest (1.7 years) in the PMs. All patients stopped tamoxifen because of the disease progression.

In Table 3, the associations between the predicted phenotypes and TTP and OS, respectively, are shown. The OS in EMs and IMs was not significantly different (HR=0.87; *P*=0.62; 95% CI: 0.50–1.50). However, OS was significantly shorter for the PMs compared with EMs (HR=2.09; *P*=0.034; 95% CI: 1.06–4.12). Although not significantly different, PMs tended to have a worse TTP (HR=1.69; *P*=0.11; 95% CI: 0.90–3.19) compared with EMs.

**Figure 1:**
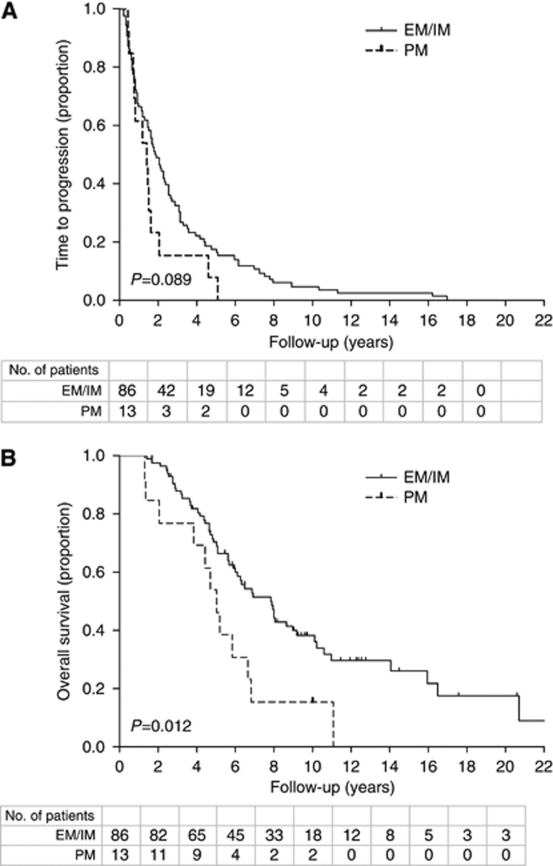
(**A**) Kaplan–Meier curves for time to progression and (**B**) overall survival, based on CYP2D6-predicted phenotype. EM, extensive metabolisers; IM, intermediate metabolisers; PM, poor metabolisers.

As intermediate and EMs had a comparable TTP and OS, both were taken together in the Kaplan–Meier estimates. The median OS-time period for PMs was 5.0 years (95% CI: 4.1–5.9) compared with 7.9 years (95% CI: 6.2–9.5) for the other predicted phenotype groups, which is statistically significantly different (*P*=0.012) (Figure 1). For PMs, the Kaplan–Meier estimate shows a non-significantly shorter median TTP compared with the combined group of other predicted phenotypes (IMs+EMs), being 1.4 years (95% CI: 0.7–2.2) *vs* 1.8 years (95% CI: 1.4–2.3), respectively, (*P*=0.089), as shown in Figure 1.

Although there was no significant association between CYP2D6 genotype and TTP or OS (HR=1.29; *P*=0.49; 95% CI: 0.63–2.66 and HR=1.38; *P*=0.42; 95% CI: 0.63–2.99, respectively), the seven patients using tamoxifen together with a CYP2D6 inhibitor had a significantly worse TTP (HR=2.97; *P*=0.008; 95% CI: 1.33–6.67) and OS (HR=3.55; *P*=0.002; 95% CI: 1.59–7.96) compared with tamoxifen users without co-administration of CYP2D6 inhibitors (Table 4).

Previous treatment, race and number of metastatic sites, as mentioned in Table 1, did not influence our model as a confounder or effect modifier.

## Discussion

In this study, we showed that MBC patients with a positive hormone receptor status treated with tamoxifen and having a CYP2D6 ‘PM-predicted phenotype’ have a statistically significant shorter OS than patients with an intermediate or extensive phenotype. In addition, we showed that patients using tamoxifen together with CYP2D6 inhibitors have a significantly shorter progression-free and worse OS compared with tamoxifen users not concomitantly using CYP2D6 inhibitors.

To our knowledge, this is the first time that the combined effect (predicted phenotype) of genotype and/or co-medication on tamoxifen treatment outcome has been investigated in the specific group of hormone-sensitive MBC patients. Besides this, the FDA approved fixed dose of tamoxifen used (40 mg per day) contributed to a homogeneous selection of patients in this study. Most studies published focused at a tamoxifen dose of 20 mg per day. In our study, EM and IM are not significantly different in terms of OS or TTP, although differences between these groups have been found in other studies when the tamoxifen dose is 20 mg per day (Goetz *et al*, 2005; Schroth *et al*, 2009). It is possible that 40 mg per day allows the IM patients to have a steady state concentration similar to EM or at least high enough to benefit from the treatment.

Previous studies have shown that adjuvant tamoxifen users with a PM status (only based on CYP2D6 genotype) have an increased risk of breast cancer recurrence and mortality (Goetz *et al*, 2005; Lim *et al*, 2007; Schroth *et al*, 2007; Bijl *et al*, 2009). In our study, no association was found between solely CYP2D6 genotype and TTP or OS in MBC patients. This may be explained by the relatively small group of CYP2D6 PMs in our set of patients. An alternative explanation can be that the effect of genotype observed in the adjuvant setting is less pronounced in MBC. On the other hand, with respect to the adjuvant setting, several other studies also did not show an association between CYP2D6 genotype and tamoxifen efficacy. In two studies on adjuvant tamoxifen treatment, no differences were found in survival rates between PMs and other metabolising groups (Nowell *et al*, 2005; Wegman *et al*, 2007). In another study, Wegman *et al* (2005)even showed a better recurrence-free survival in PMs. The conflicting results may partly be explained by differences or heterogeneity in study populations and in view of our data also by lack of information about concomitant use of CYP2D6 inhibitors and hormone receptor status. Differences in dysfunctional alleles that have been studied also make it difficult to compare the results and can cause misinterpretation (Dezentje *et al*, 2009). The gene encoding for CYP2D6 is highly polymorphic, with CYP2D6*4 being the most common dysfunctional variant allele in Caucasians [5]. In our study, besides CYP2D6 *4, also the CYP2D6 dysfunctional alleles CYP2D6*3, *5, *6, *10 and *41 having a low allele frequency, have been included in the analysis (Bradford, 2002).

Retrospective analyses clearly have shortcomings (that is, incomplete information on breast cancer stage, tumour size or nodal stage, or treatment given after progression on tamoxifen), but for this study there is no reason to assume that these variables differ significantly between the three phenotype groups nor have influenced outcome (Schroth *et al*, 2007). In addition, information about compliance may not have been complete as this was gathered retrospectively from patient charts. In a study by Rae *et al* (2009), an association between CYP2D6 genotype and compliance to tamoxifen therapy was shown, as PMs were more likely to continue tamoxifen therapy compared with intermediate or EMs. The results suggest that EMs are more likely to both obtain benefit from tamoxifen therapy and discontinue the drug because of side effects (Rae *et al*, 2009). This implies that the observed effects of tamoxifen in our study may even be underestimated by decreased adherence or persistence to therapy. Regarding the inclusion criteria there may have been selection bias, as not all patients may have been included. In that case we would expect to now have fewer PMs in the analysis compared with IMs or EMs, as they have a shorter OS and may have died before inclusion took place. This would also mean that our data may underestimate the effect described.

Our results are in line with the findings by Kelly *et al* (2010), who also found that tamoxifen may be less effective in patients treated with tamoxifen while taking CYP2D6 inhibitors, although the number of patients using this co-medication was relatively small in our study group. Nevertheless, it is striking to notice that relatively large differences in therapeutic outcome were found. For treatment of hot flushes as a side effect of tamoxifen therapy SSRIs are often prescribed (Goetz and Loprinzi, 2003). Clearly the potential negative effect of such SSRIs on the efficacy of the anti-cancer effect of tamoxifen is a matter of great concern. We could not ascertain the indication for anti-depressant treatment to rule out that the underlying disorder is responsible for the effect seen. But as the findings by Kelly *et al* (2010) show an increased mortality risk only with potent and not with weak or moderate inhibitors of CYP2D6 showing a strong biological plausibility, selection bias is not readily suggested. However, this will have to be confirmed in prospective studies.

Although further research is needed, this may imply that CYP2D6 EMs can preferentially be treated with tamoxifen instead of aromatase inhibitors. On the other hand, CYP2D6 PMs and patients who cannot avoid using CYP2D6-inhibiting medication might benefit more from aromatase inhibitors over tamoxifen, as these compounds have another metabolic route. Another option might be to give an escalated tamoxifen dose to increase plasma endoxifen concentrations. Tamoxifen doses higher than 40 mg daily may theoretically be necessary to achieve adequate plasma concentrations of active tamoxifen metabolites in patients with a PM phenotype.

On the basis of our study and the current literature, we would favour to study not only CYP2D6 genotype but to focus more on CYP2D6 phenotype, thereby taking co-medication into account. One way to do this is by giving the patient a CYP2D6 activity probe drug that mimics the metabolism of tamoxifen (Mathijssen and van Schaik, 2006). Currently, such a phenotyping study is ongoing at our clinic (see http://www.trialregister.nl: NTR number 1751). In addition, it would be of interest to further explore the role of plasma concentrations of tamoxifen metabolites (that is, endoxifen) as predictors of therapeutic outcome (Kiyotani *et al*, 2010). Therapeutic drug monitoring should therefore be included in prospective studies that evaluate the effect of CYP2D6 phenotype on tamoxifen treatment outcome.

In summary, we showed that CYP2D6-predicted phenotype, described as the combined effect of CYP2D6 genotype and co-prescribed CYP2D6-inhibiting medication, is a predictor of outcome in women using tamoxifen (40 mg per day) for the treatment of hormone receptor-positive MBC. As co-administration of CYP2D6-inhibiting medication seems to diminish the treatment effect of tamoxifen, this combination of drugs should be handled with care.

## Figures and Tables

**Table 1:** Baseline Characteristics

**Variable**	**Number of patients (%) *n*=102**
Age first tamoxifen use for metastatic breast cancer, average (s.d.)	51.8 (9.1) years
	
*Race*	
Caucasian	97 (95.1)
Asian	4 (3.9)
African	1 (1.0)
	
*Previous treatments*	
Operative procedure	
Mastectomy	41 (40.2)
Lumpectomy	53 (52.0)
None	8 (7.8)
Previous adjuvant therapy	
Radiotherapy	33 (32.3)
Chemotherapy	22 (21.6)
Both chemotherapy and radiotherapy	21 (20.6)
None	26 (25.5)
Previous therapy for metastatic disease	
Radiotherapy	17 (16.6)
Chemotherapy	21 (20.6)
Both chemotherapy and radiotherapy	11 (10.8)
None	53 (52.0)
	
*No. of metastatic sites*	
1	69 (67.6)
2	27 (26.5)
3	6 (5.9)
	
*Metastatic site*	
Lymph	27 (19.1)
Bone	62 (44.0)
Lung	30 (21.3)
Liver	13 (9.2)
Skin	8 (5.7)
Other	1 (0.7)
	
*CYP2D6 genotypes*	
wt/wt	45 (44.1)
wt/*3	2 (2.0)
*3/*3	0 (0.0)
*3/*4	4 (3.9)
*3/*41	1 (1.0)
wt/*4	25 (24.5)
*4/*4	4 (3.9)
*4/*6	1 (1.0)
*4/*10	1 (1.0)
*4/*41	4 (3.9)
wt/*5	3 (2.9)
*5/*5	0 (0.0)
wt/*6	1 (1.0)
*6/*6	0 (0.0)
wt/*10	2 (2.0)
wt/*41	6 (5.9)
Unknown	3 (2.9)
	
*CYP2D6-inhibiting co-medication*	7 (6.9)
Strong	5 (4.9)^a^
Moderate	0 (0.0)
Weak	2 (2.0)^b^

Abbreviations: CYP2D6=cytochrome P450 2D6; wt=wild type.

a Paroxetine (*n*=4), fluoxetine (*n*=1).

b Citalopram (*n*=1), sertraline (*n*=1).

**Table 2:** CYP2D6-predicted phenotype based on the combination of the CYP2D6 genotype and concomitant use of a CYP2D6 inhibitor

**CYP2D6 phenotype** a	**CYP2D6 genotype**	**Strong inhibitor**	**Moderate inhibitor**	**Weak inhibitor**	***N*=99 (%)**
EM	wt/wt	No	No	No	41 (41.4)
	wt/*10	No	No	No	2 (2.0)
	wt/*41	No	No	No	5 (5.1)
IM	wt/*3	No	No	No	1 (1.0)
	wt/*4	No	No	No	25 (25.3)
	wt/*5	No	No	No	3 (3.0)
	wt/*6	No	No	No	1 (1.0)
	*3/*41	No	No	No	1 (1.0)
	*4/*10	No	No	No	1 (1.0)
	*4/*41	No	No	No	4 (4.0)
	wt/wt	No	No	Yes	2 (2.0)
PM	*3/*4	No	No	No	3 (3.0)
	*3/*4	Yes	No	No	1 (1.0)
	*4/*4	No	No	No	4 (4.0)
	*4/*6	No	No	No	1 (1.0)
	wt/*3	Yes	No	No	1 (1.0)
	wt/*41	Yes	No	No	1 (1.0)
	wt/wt	Yes	No	No	2 (2.0)

Abbreviations: CYP2D6=cytochrome P450 2D6; EM=extensive metabolisers; IM=intermediate metabolisers; PM=poor metabolisers; wt=wild type.

a On the basis of CYP2D6 genotype (*CYP*2D6*3, *4, *5, *6, *10 and *41) and concomitant use of CYP2D6-inhibiting medication.

**Table 3:** Association between CYP2D6 predicted phenotype, time to progression and overall survival

	**Time to progression**			**Overall survival**		
**CYP2D6 phenotype** a	**Cases (*n*=99)**	**HR**^b^ **(95% CI)**	***P*-value**	**Cases (*n*=67)**	**HR**^b^ **(95% CI)**	***P*-value**
EM	48	1.00 (ref)		33	1.00 (ref)	
IM	38	0.99 (0.64–1.55)	0.98	22	0.87 (0.50–1.50)	0.62
PM	13	1.69 (0.90–3.19)	0.11	12	2.09 (1.06–4.12)	0.03

Abbreviations: CI=confidence interval; CYP2D6=cytochrome P450 2D6; EM=extensive metabolisers; HR=hazards ratio; IM=intermediate metabolisers; PM=poor metabolisers; ref=reference.

a On the basis of CYP2D6 genotype (*CYP*2D6*3, *4, *5, *6, *10 and *41) and concomitant use of CYP2D6-inhibiting medication.

b HRs were calculated using Cox-proportional hazards models and were adjusted for age at the index date.

**Table 4:** Association between CYP2D6-inhibiting co-medication, time to progression and overall survival

	**Time to progression**			**Overall survival**		
**CYP2D6-inhibiting Co-medication** a	**Cases (*n*=102)**	**HR**^b^ **(95% CI)**	***P*-value**	**Cases (*n*=70)**	**HR**^b^ **(95% CI)**	***P*-value**
No	95	1.00 (ref)		63	1.00 (ref)	
Yes	7	2.97 (1.33–6.67)	0.008	7	3.55 (1.59–7.96)	0.002

Abbreviations: CI=confidence interval; CYP2D6=cytochrome P450 2D6; HR=hazards ratio; ref=reference.

a Co-medication: paroxetine (*n*=4), fluoxetine (*n*=1), sertraline (*n*=1) and citalopram (*n*=1).

b HRs were calculated using Cox-proportional hazards models and were adjusted for genotype (PM, IM and EM based on *CYP*2D6*3, *4, *5 ,*6, *10 and *41) and age at the index date.
